# Characterization of nonstructural protein 3 of a neurovirulent Japanese encephalitis virus strain isolated from a pig

**DOI:** 10.1186/1743-422X-8-209

**Published:** 2011-05-09

**Authors:** Xufang Deng, Zixue Shi, Shuqing Li, Xiaodu Wang, Yafeng Qiu, Donghua Shao, Jianchao Wei, Guangzhi Tong, Zhiyong Ma

**Affiliations:** 1 Shanghai Veterinary Research Institute, Chinese Academy of Agricultural Science, No. 518, Ziyue Road, Shanghai, 200241, PR China; 2 Shanghai Entry-Exit Inspection and Quarantine Bureau, No. 1208, Min sheng Road, Shanghai, 200135, PR China; 3 Forestry and Biotechnology School, Zhejiang Agriculture and Forestry University, Lin'an, Hangzhou, 311300, PR China

## Abstract

**Background:**

Japanese encephalitis virus (JEV), as a re-emerging virus that causes 10,000-15,000 human deaths from encephalitis in the world each year, has had a significant impact on public health. Pigs are the natural reservoirs of JEV and play an important role in the amplification, dispersal and epidemiology of JEV. The nonstructural protein 3 (NS3) of JEV possesses enzymatic activities of serine protease, helicase and nucleoside 5'-triphosphatase, and plays important roles in viral replication and pathogenesis.

**Results:**

We characterized the NS3 protein of a neurovirulent strain of JEV (SH-JEV01) isolated from a field-infected pig. The NS3 gene of the JEV SH-JEV01 strain is 1857 bp in length and encodes protein of approximately 72 kDa with 99% amino acid sequence identity to that of the representative immunotype strain JaGAr 01. The NS3 protein was detectable 12 h post-infection in a mouse neuroblastoma cell line, Neuro-2a, and was distributed in the cytoplasm of cells infected with the SH-JEV01 strain of JEV. In the brain of mice infected with the SH-JEV01 strain of JEV, NS3 was detected in the cytoplasm of neuronal cells, including pyramidal neurons of the cerebrum, granule cells, small cells and Purkinje cells of the cerebellum.

**Conclusions:**

The NS3 protein of a neurovirulent strain of JEV isolated from a pig was characterized. It is an approximately 72 kDa protein and distributed in the cytoplasm of infected cells. The Purkinje cell of the cerebellum is one of the target cells of JEV infection. Our data should provide some basic information for the study of the role of NS3 in the pathogenesis of JEV and the immune response.

## Background

Japanese encephalitis (JE), known previously as Japanese B encephalitis, is caused by Japanese encephalitis virus (JEV). JE is most prevalent in Southeast Asia and the Far East, and causes 10,000-15,000 human deaths from encephalitis in the world each year [1]. JEV is a mosquito-borne virus of the *Flavivirus *genus in the family *Flaviviridae*. Its genome is positive-sense single-stranded RNA, approximately 11 kb in length, which encodes a precursor polyprotein consisting of three structural proteins (C, prM and E) and seven nonstructural proteins (NS1, NS2A, NS2B, NS3, NS4A, NS4B and NS5) [2].

The nonstructural protein 3 (NS3) of JEV is a multifunctional protein of 619 amino acid residues. It possesses enzymatic activities of serine protease, helicase and nucleoside 5'-triphosphatase, and plays important roles in the processing of the viral precursor polyprotein and the replication of viral genomic RNA [3]. In JEV-infected cells, NS3 is associated with microtubules and tumor susceptibility gene 101 protein, which play essential roles in viral RNA packaging, intracellular trafficking of viral components and viral assembly [4-6]. NS3 is localized mainly at the JEV-induced convoluted membrane, a membrane vesicle structure, which has been proposed to originate from rough endoplasmic reticulum, Golgi apparatus or the trans-Golgi network, and serves as a reservoir for viral proteins during virus assembly [6]. Therefore, NS3 has been considered to be a candidate target molecule for development of novel potent therapeutic substances [7].

In addition to humans, other mammalian animals and birds are susceptible to JEV. Infection with JEV causes reproductive disorders, such as orchitis, stillbirths and mummified fetuses, in breeding pigs and encephalitis in piglets, but no observable clinical signs in growing and adult pigs. The JEV-infected pigs develop a level of viremia that remains high enough to infect mosquitoes for up to 4 days [8]. Pigs are the major amplifying hosts of JEV, and also act as maintenance hosts in endemic areas [9]. Despite the fact that pigs play an important role in the amplification, dispersal and epidemiology of JEV, JEV strains isolated from pigs have not been studied thoroughly.

In this study, we characterized the NS3 protein of a neurovirulent strain of JEV (SH-JEV01) isolated from a field-infected pig, because NS3 is a multifunctional protein that plays important roles not only in viral replication, as described above, but also in the host immune response and pathogenesis. Previous studies have demonstrated that NS3 is a dominant CD4+ as well as CD8+ T cell-eliciting antigen [10] and is able to induce caspase 3 activation and mitochondria-mediated apoptosis in human medulloblastoma cells [11]. The characterization of the NS3 protein of the JEV strain isolated from pigs should provide some basic information for the study of its role in the pathogenesis of JEV and the immune response.

## Results

### Cloning and expression of the NS3 gene

**Figure 1:**
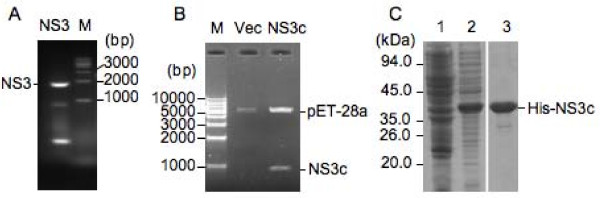
**Cloning and expression of the NS3 gene**. (A) NS3 gene was amplified from JEV-infected cells by RT-PCR and checked by agarose gel electrophoresis. Lane M, DNA ladder. (B) The recombinant plasmid expressing the C-terminal region of the NS3 protein (NS3c) was double digested with EcoR I and Sal I and separated by agarose gel electrophoresis. Lane M, DNA ladder; Lane Vec, pET-28a empty vector; Lane NS3c, recombinant plasmid expressing NS3c protein. (C) Analysis of the expression and purification of His-tagged NS3c (His-NS3c). Bacterial lysates and purified His-NS3c were separated on a 10% SDS-PAGE gel and stained with Coomassie blue R250. Lane 1, uninduced bacterial cells; lane 2, bacterial cells induced with isopropyl-β-D-thiogalactopyranoside (IPTG); Lane 3, His-NS3c eluted from the His Bind Resin column.

The gene encoding the full-length NS3 protein of JEV SH-JEV01 strain was amplified from JEV-infected cells by reverse transcription-polymerase chain reaction (RT-PCR). The amplified products were 1857 bp in length (Figure 1A) and confirmed by DNA sequencing. The amino acid sequence deduced from the nucleotide sequence of NS3 consists of 619 amino acid residues and showed 99% identity to that of the representative immunotype strain JaGAr 01 (GenBank No: AF069076). To express a truncated NS3 protein in prokaryotic cells for generation of antibodies against NS3, a segment encoding the C-terminal region of NS3 (NS3c) was sub-cloned into the expression vector pET-28a (Figure 1B). The NS3c protein was expressed in *Escherichia coli *(Figure 1C, lane 2) and purified by Ni^2+ ^affinity chromatography using Ni-NTA beads. Peak A_280 _fractions eluted from the column were pooled and showed a predominant single His-NS3c band (Figure 1C, lane 3).

### Generation of polyclonal antibodies against NS3

**Figure 2:**
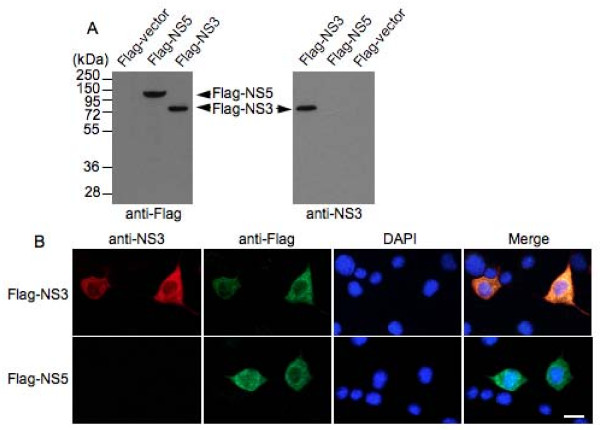
**Analysis of the specificity of the anti-NS3 antibody produced**. (A) Western blot analysis of Flag-NS3 expressed in Neuro-2a cells. Cell lysates (20 μg) from Neuro-2a cells transfected with plasmid expressing Flag-NS3 were probed with the anti-NS3 antibody (anti-NS3 panel) and then re-probed with anti-Flag antibody (M2, Sigma) (anti-Flag panel). The plasmid expressing Flag-tagged JEV NS5 protein (Flag-NS5) and Flag-empty vector (Flag-vector) were used as controls. (B) Immunofluorescence analysis of Flag-NS3 expressed in Neuro-2a cells. Neuro-2a cells transfected with plasmid expressing Flag-NS3 were double-immunostained with the anti-NS3 antibody (anti-NS3 panel, red) and anti-Flag antibody (anti-Flag panel, green). The cells transfected with plasmid expressing Flag-tagged JEV NS5 protein (Flag-NS5) were used as controls. The cells were also stained for DNA with 4', 6'-diamidino-2-phenylindole (DAPI) (DAPI panel, blue). Merge panel shows the superimposed image. Bar, 20 μm.

The purified His-NS3c was injected into mice for production of antibodies. The titer of the antiserum collected at 7 days after the fourth immunization was determined by enzyme-linked immunosorbent assay (ELISA) (data not shown). The antiserum with the highest ELISA titer (1:128,000) was temporarily designated "anti-NS3 antibody" and used for subsequent experiments. To test the specificity of the anti-NS3 antibody, Neuro-2a cells were transfected transiently with the plasmid Flag-NS3 and subjected to western blot and immunofluorescence analysis using the anti-NS3 antibody. A plasmid expressing the Flag-tagged NS5 protein of JEV (Flag-NS5) was used as the control. The western blot analysis revealed that the anti-NS3 antibody reacted specifically with the Flag-NS3 protein expressed in Neuro-2a cells (Figure 2A). The immunofluorescence analysis demonstrated that the anti-NS3 antibody stained the Neuro-2a cells transfected with the plasmid Flag-NS3 (Figure 2B). Taken together, these results indicated that the anti-NS3 antibody is highly specific and is able to detect the NS3 protein.

### Expression of NS3 in JEV-infected cells and mouse brains

**Figure 3:**
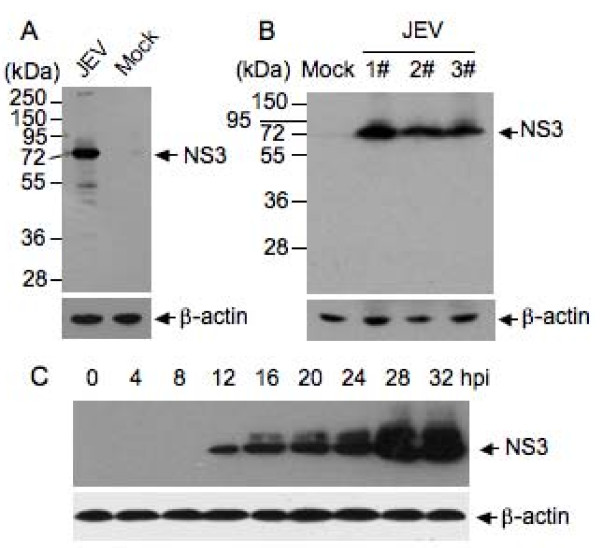
**Detection of the expression of NS3 by western blot analysis**. The lysates from JEV-infected cells or mouse brains were immunoblotted with the anti-NS3 antibody and re-probed with anti-β-actin antibody (AC-15, Sigma). (A) Neuro-2a cells were infected with the SH-JEV01 strain of JEV at MOI 5 and analyzed 12 h post-infection (hpi). Mock-infected cells (Mock) were used as controls. (B) Brain homogenates from mice infected with the SH-JEV01 strain of JEV (1.0 × 10^6 ^PFU) were analyzed by western blot. Brain homogenates from the mock-infected mice (Mock) were used as controls. Lane 1#, 2# and 3#, number of JEV-infected individual mice. (C) Neuro-2a cells were infected with the SH-JEV01 strain of JEV at MOI 5 and analyzed at the indicated times.

The mouse neuroblastoma cell line Neuro-2a has been used for JEV studies [12,13], so we used Neuro-2a cells for analysis of NS3 expression by western blot. The NS3 protein expressed in cells infected with the SH-JEV01 strain of JEV has a molecular mass of approximately 72 kDa (Figure 3A), which is similar to that of the RP9 strain [6]. We also detected the NS3 protein in the brain homogenates of mice infected with the SH-JEV01 strain of JEV. A single band of 72 kDa corresponding to NS3 was detected (Figure 3B). The dynamics of NS3 expression during infection of Neuro-2a cells were determined at various times post-infection (hpi) by western blot analysis; as shown in Figure 3C, the expression of NS3 was detectable from 12 hpi with a gradually increasing pattern.

### Intracellular localization of NS3 in JEV-infected cells

**Figure 4:**
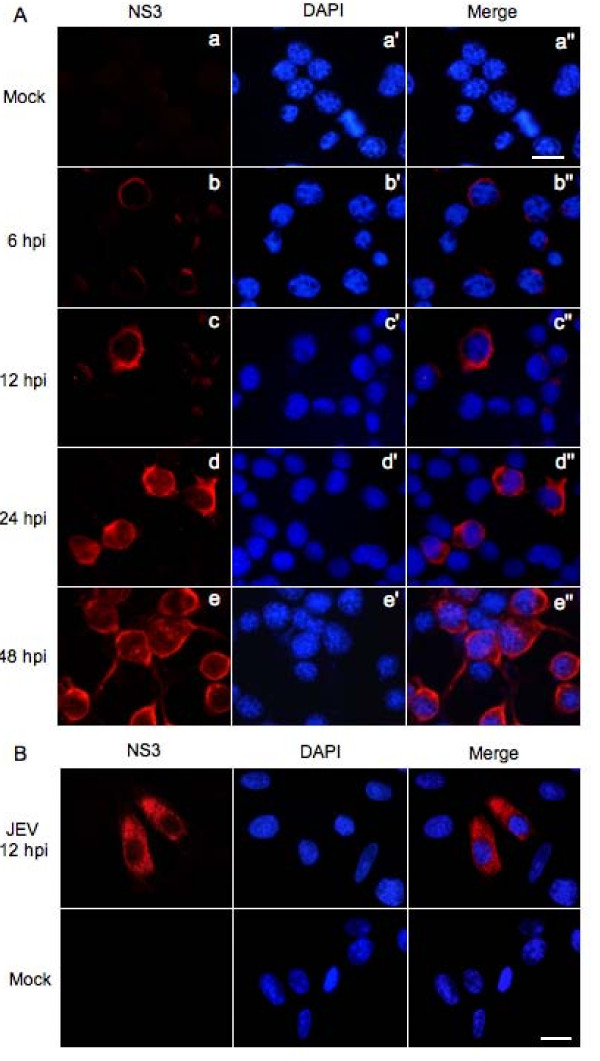
**Analysis of the intracellular localization of NS3**. Neuro-2a (A) and PIEC cells (B) were infected with the SH-JEV01 strain of JEV and immunostained with anti-NS3 antibody (red) at the indicated times. Mock-infected cells (Mock) were used as controls. The cells were also stained for DNA with 4', 6'-diamidino-2-phenylindole (DAPI) (blue). Merge panel shows the superimposed image. hpi, hours post-infection. Bar, 20 μm.

To detect the intracellular localization of NS3, Neuro-2a cells were infected with the SH-JEV01 strain of JEV and the intracellular localization of NS3 was detected at various times post-infection by immunofluorescence analysis. As shown in Figure 4A, the NS3 protein, shown as red fluorescence, appeared from 6 hpi at the perinuclear cytoplasm region of JEV-infected cells (panel b). The NS3 staining increased in intensity and emanated into the cytoplasm with increased time post-infection (panels c and d). At 48 hpi, NS3 was distributed widely in the cytoplasm, including the neurodendrites (panel e). No significant fluorescence was observed in mock-infected cells (panel a). The cytoplasmic distribution of the NS3 was also observed in porcine vascular endothelial cells (PIEC) infected with the SH-JEV01 strain of JEV (Figure 4B).

### Localization of NS3 in mouse brain cells

**Figure 5:**
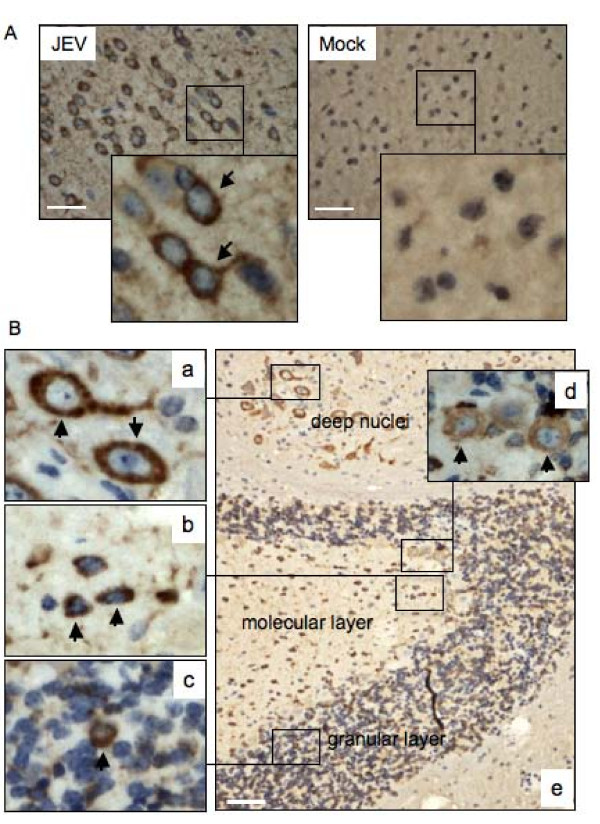
**Detection of NS3 in mouse brain cells**. One-week-old sucking mice were infected with the SH-JEV01 strain of JEV (1.0 × 10^6 ^PFU). Brain samples were collected from mice showing neurological signs and stained immunohistochemically with the anti-NS3 antibody. The nuclei were counter-stained by hematoxylin. Brain samples from the mock-infected mice (Mock) were used as controls. (A) Immunohistochemical examination of the cerebral cortex. Bar, 50 μm. (B) Immunohistochemical examination of the cerebellum. Bar, 200 μm. Arrows indicate the NS3-positive cells.

To detect the localization of NS3 in brain cells of mice infected with the SH-JEV01 strain of JEV, brain sections from JEV-infected mice were stained immunohistochemically with the anti-NS3 antibody. The nuclei were counter-stained with hematoxylin, which shows light or dark blue. The NS3 protein, shown as brown deposits by immunoperoxidase stains, was found to be distributed in the cytoplasm of neuronal cells in the cerebrum; a higher NS3-positive rate was detected in the pyramidal neurons of the cerebral cortex (Figure 5A, JEV panel). The neuronal cells of the mock-infected mice did not show visible staining for NS3 protein (Figure 5A, Mock panel). The NS3 protein was not detectable in the cerebellum of the mock-infected mice (data not shown). In contrast, NS3 protein was found in the cytoplasm of neuronal cells of the deep nuclei (panel a), small cells of the molecular layer (panel b) and granule cells of the granular layer (panel c) of the cerebellum of JEV-infected mice (Figure 5B).

### Detection of NS3 in Purkinje cells

**Figure 6:**
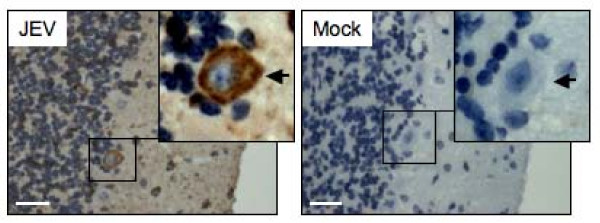
**Detection of NS3 in Purkinje cells**. One-week-old sucking mice were infected with the SH-JEV01 strain of JEV (1.0 × 10^6 ^PFU). Samples were collected from the cerebellum of mice showing the neurological signs and stained immunohistochemically with the anti-NS3 antibody. The nuclei were counter-stained by hematoxylin. Cerebellar samples from the mock-infected mice (Mock) were used as controls. Arrows indicate Purkinje cells. Bar, 50 μm.

Purkinje cells are long-axon neurons in the cerebellum and function as the sole output cells of the cerebellar cortex. Abnormalities in cerebellar Purkinje cells result in neurological signs such as ataxia [14]. The mice infected with the SH-JEV01 strain of JEV showed clinical signs of ataxia in our experiments (data not shown); we therefore detected the NS3 protein in Purkinje cells using immunohistochemical analysis. The NS3 staining was detected in the cytoplasm of certain Purkinje cells of JEV-infected mice, with a positive rate of 15.3% (Figure 6, JEV panel; Figure 5B, panel d), but not in the cells of the mock-infected mice (Figure 6, Mock panel), which suggests that Purkinje cells are infected by JEV.

## Discussion

Pigs are the natural reservoirs of JEV, and JEV-infected pigs act as important amplifying hosts of JEV [15]. Pigs infected with JEV develop a level of viremia that makes the virus available to mosquitoes [8]. Despite the fact that pigs play an important role in the amplification, dispersal and epidemiology of JEV, JEV strains isolated from pigs have not been studied thoroughly. In this study, we used the mouse neuroblastoma cell line Neuro-2a and mouse models to characterize the NS3 protein, a multifunctional protein that plays important roles in viral replication and pathogenesis, of a neurovirulent strain of JEV (SH-JEV01) isolated from a field-infected pig.

The NS3 gene of the SH-JEV01 strain of JEV is 1857 bp in length (Figure 1A). The amino acid sequence deduced from the nucleotide sequence of NS3 consists of 619 amino acid residues and shows 99% identity to that of the representative immunotype JaGAr 01 strain (GenBank No: AF069076). The JaGAr 01 strain was isolated from *Culex *mosquitoes in Japan in 1959 and is neurovirulent and neuroinvasive [16]. The molecular mass of the NS3 of the SH-JEV01 strain of JEV was approximately 72 kDa, as measured by western blot analysis (Figure 3A and 3B), which is similar to that of the RP9 strain [6]. The NS3 protein was detectable 12 hpi in Neuro-2a cells and was distributed in the cytoplasm of infected cells (Figure 4), which is in agreement with previous studies [4,6]. In the brain of JEV-infected mice, NS3 was detected in the cytoplasm of neuronal cells, including pyramidal neurons of the cerebrum (Figure 5), granule cells, small cells and Purkinje cells of the cerebellum (Figure 5 and 6), which is similar to the findings of previous studies [4] and suggested that the SH-JEV01 strain of JEV is neurovirulent and neuroinvasive in sucking mouse.

Cerebellar Purkinje cells are critical for the normal function of the cerebellum. Abnormalities in cerebellar Purkinje cells result in neurological signs such as ataxia [15]. We found that 15.3% of cerebellar Purkinje cells were positive for NS3 staining (Figure 5B, panel d and Figure 6), which suggests that the Purkinje cell is one of the target cells of JEV infection. However, whether JEV infects cerebellar Purkinje cells is disputed, because Wang *et al. *[4] and Johnson *et al. *[17] demonstrated no viral antigens in cerebellar Purkinje cells, whereas Desai *et al. *[18] found that viral antigens were present in Purkinje cells. This controversy is presumably due to the differences in virulence and cell tropism between JEV strains. The JEV strain that did not infect cerebellar Purkinje cells in the study of Wang *et al. *[4] is a mutant strain developed by γ-ray irradiation, which mainly targets the cerebrum rather than the cerebellum [4]. In contrast, the JEV stain used in our study is a neurovirulent strain, which causes severe signs of encephalitis, including ataxia, in the mouse. Purkinje cells are the major output neurons of the cerebellar cortex and provide inhibitory feedback into the deep cerebellar nuclei [15]. Our data indicated that the Purkinje cells were targeted by JEV, suggesting a potential role of the infected Purkinje cells in JEV neuropathogenesis. However, the significance of JEV-infected Purkinje cells in the viral neuropathogenesis needs to be further explored.

NS3 has been described to be involved in the activation of caspase 3 and mitochondria-mediated apoptosis [11]. We attempted to determine whether NS3 is localized in mitochondria, which play an important role in the regulation of apoptosis, by immunofluorescence analysis. In the JEV-infected Neuro-2a cells, the co-localization of NS3 and GRP75, a mitochondrial protein [19], was not observed (data not shown), which implies that NS3 does not localize in mitochondria. In addition, no NS3 protein was detected in the hearts, kidneys and testes that were collected from the JEV SH-JEV01-infected mice at necropsy (data not shown), although the mice had been inoculated intraperitoneally with JEV.

## Conclusions

In conclusion, we characterized the NS3 protein of a neurovirulent strain of JEV (SH-JEV01) isolated from a field-infected pig. The NS3 gene of the JEV SH-JEV01 strain is 1857 bp in length and encodes protein of approximately 72 kDa. The NS3 protein was detectable 12 h post-infection in a mouse neuroblastoma cells, and was distributed in the cytoplasm of cells infected with the SH-JEV01 strain of JEV. In the brain of mice infected with the SH-JEV01 strain of JEV, NS3 was detected in the cytoplasm of neuronal cells, including pyramidal neurons of the cerebrum, granule cells, small cells and Purkinje cells of the cerebellum. Our data should provide some basic information for the study of its role in the pathogenesis of JEV and the immune response.

## Methods

### Virus and cells

A neurovirulent strain of JEV (SH-JEV01), isolated from aborted fetuses of sow, was grown in 3-day-old BALB/c mice and titrated by plaque assay using a baby-hamster kidney cell line (BHK-21). A mouse neuroblastoma cell line, Neuro-2a (ATCC No. CCL-131), PIEC and BHK-21 cells were grown in Dulbecco's modified Eagle's medium (DMEM) supplemented with 10% fetal bovine serum (FBS) at 37°C in an atmosphere containing 5% CO_2_.

### Viral infection

Neuro-2a, PIEC and BHK-21 cells were pre-cultured on 6-well plates overnight. The cells were inoculated with the SH-JEV01 strain of JEV at a multiplicity of infection (MOI) of 5 when the cells reached approximately 50-70% confluence. The inoculum was removed following a 1.5 h period of incubation, and the cells were further incubated in DMEM with 1% FBS for the indicated times.

### Cloning and expression

A sequence encoding the full length of the JEV NS3 protein was amplified from BHK-21 cells infected with the SH-JEV01 strain of JEV by reverse transcription (RT) using 6 bp random primers and polymerase chain reaction (PCR) with NS3 specific primers (forward 5'-ATGAATTCAGGGGGCGTGTTTTG-3', reverse 5'-GCGTCGACTTATCTCTTCCCTGCTGCA-3'). The RT-PCR product was analyzed by DNA sequencing, and was inserted subsequently into the EcoR I-Sal I cloning site of a eukaryotic expression vector, p3xFLAG-CMV-7.1 (Sigma, St. Louis, MO, USA) to generate the recombinant plasmid Flag-NS3. Transfection of plasmid Flag-NS3 into Neuro-2a cells was performed using Lipofectamine™ 2000 (Invitrogen, Carlsbad, CA, USA), according to the manufacturer's protocol. The expression of NS3 in the transfectants was determined by western blot and immunofluorescence analysis.

To obtain an immunogen for the preparation of antibodies against NS3, a truncated sequence (955 bp to 1857 bp) encoding the C-terminal region of the NS3 protein (NS3c) was amplified from plasmid Flag-NS3 by PCR using the forward primer 5'-ATGAATTCATGCCGCCTGGAACCACGG-3', and the reverse primer 5'- GCGTCGACTTATCTCTTCCCTGCTGCA-3'. The amplified NS3c fragment was sub-cloned into the EcoR I-Sal I cloning site of a prokaryotic expression vector pET-28a (Novagen, Madison, WI, USA) in order to express the histidine (His)-tagged NS3c (His-NS3c) heterologously in *Escherichia coli *strain BL21 (DE3). The expression was induced by treatment with 1 mM isopropyl-β-D-thiogalactopyranoside (IPTG) at 37°C for 5 h, and the expression product was purified using a His Band kit (Novagen), according to the manufacturer's instructions. The purity of the preparation was assessed by 10% sodium dodecyl sulfate polyacrylamide gel electrophoresis (SDS-PAGE) followed by staining with Coomassie blue R250.

### Generation of polyclonal antibody

To prepare polyclonal antibodies against NS3, 6-week-old ICR mice (n = 5) were immunized intraperitoneally with 50 μg purified His-NS3c fusion protein emulsified in complete Freund's adjuvant (Sigma). Subsequent booster immunizations of 50 μg purified His-NS3c in incomplete Freund's adjuvant (Sigma) were given intraperitoneally to each mouse on days 14, 28, and 42. The mice were bled 7 days after the fourth immunization. The titer of the antiserum was tested by ELISA. The specificity of the antiserum was determined by western blot and immunofluorescence analysis. The animal experiments were in compliance with the Guidelines on the Humane Treatment of Laboratory Animals (Ministry of Science and Technology of the People's Republic of China, Policy No. 2006 398) and were approved by the Institutional Animal Care and Use Committee at the Shanghai Veterinary Research Institute, Chinese Academy of Agricultural Science.

### Infection of JEV into mice

Three C57B/L6 sucking mice (1 week old) were inoculated intraperitoneally with 0.1 ml of the SH-JEV01 strain of JEV (1.0 × 10^6 ^PFU). The mice showed neurological signs such as ataxia 4 days after inoculation. The mice that showed neurological signs were euthanized according to the Guidelines on the Humane Treatment of Laboratory Animals (Policy No. 2006 398). For immunohistochemical examination, the brains of the mice were collected and fixed in 10% neutral buffered formalin. For western blot analysis, small pieces of mouse brain were homogenized in a lysis buffer (20 mM Tris-HCl pH 7.5, 150 mM NaCl, 1 mM Na2-EDTA, 1% Triton-100, 1 μg/ml leueptin, 1 μg/ml aprotinin, 1 mM PMSF). Two mice inoculated intraperitoneally with an equivalent volume of phosphate-buffered saline were used as controls. The animal experiments were in compliance with the Guidelines on the Humane Treatment of Laboratory Animals (Policy No. 2006 398) and were approved by the Institutional Animal Care and Use Committee at the Shanghai Veterinary Research Institute, Chinese Academy of Agricultural Science.

### Immunohistochemistry

Mouse brain samples fixed in 10% neutral buffered formalin were processed routinely for preparation of paraffin wax-embedded sections. The sections were stained with primary antibody against NS3 for 30 min, and incubated subsequently with a horseradish peroxidase-conjugated goat anti-mouse IgG antibody (sc-2005, Santa Cruz Biotechnology, Santa Cruz, CA, USA) for 30 min. The sections were developed with 3, 3-diaminobenzidine (DAB) and examined with a light microscope. The nuclei were counter-stained by hematoxylin.

### ELISA

A microtiter plate was coated with 2 μg/ml of the purified His-NS3c fusion protein in carbonate-buffered saline and incubated overnight at 4°C. The plate was then washed with phosphate-buffered saline (PBS) and blocked with 1% bovine serum albumin (Sigma) in PBS. After incubation, 100 μl of antiserum at different dilutions were added to the appropriate wells and incubated for 1 h at 37°C. The plate was washed and incubated for 1 h with the horseradish peroxidase-conjugated goat anti-mouse IgG antibody (Santa Cruz) at 37°C. The reaction was initiated by adding o-phenylenediamine substrate solution (Sigma), and the absorbance at 490 nm was determined using a bichromatic microplate reader.

### Western blot and immunofluorescence analysis

Western blot and immunofluorescence analysis were performed as described previously [20].

## Competing interests

The authors declare that they have no competing interests.

## Authors' contributions

XFD carried out most of the experiments and wrote the manuscript. ZXS and SQL constructed the experimental plasmids. XDW and YFQ helped in the generation of polyclonal antibody. DHS and JCW helped in the mouse infection. GZT revised the experimental design. ZYM designed the experiments and revised the manuscript. All of the authors read and approved the final version of this manuscript.
